# A painful lesson from the COVID-19 pandemic: the need for broad-spectrum, host-directed antivirals

**DOI:** 10.1186/s12967-020-02476-9

**Published:** 2020-10-15

**Authors:** Vipul C. Chitalia, Ali H. Munawar

**Affiliations:** 1 Boston University Medical Center, 625 Albany Street, Boston, MA USA; 2 Institute for Medical Engineering & Science, Massachusetts Institute of Technology, Cambridge, MA USA; 3 Bisect Therapeutics, Inc., 45 Dan Road, Canton, MA USA

**Keywords:** COVID-19, Broad-spectrum antivirals, Mechanism of action (MOA), Pandemics, Drug discovery and development, SARS-CoV-2, Host-directed antivirals, Antiviral drug design, Coronavirus (CoV), Drug design strategies, Prophylactic antiviral therapy

## Abstract

While the COVID-19 pandemic has spurred intense research and collaborative discovery worldwide, the development of a safe, effective, and targeted antiviral from the ground up is time intensive. Therefore, most antiviral discovery efforts are focused on the re-purposing of clinical stage or approved drugs. While emerging data on drugs undergoing COVID-19 repurpose are intriguing, there is an undeniable need to develop broad-spectrum antivirals to prevent future viral pandemics of unknown origin. The ideal drug to curtail rapid viral spread would be a broad-acting agent with activity against a wide range of viruses. Such a drug would work by modulating host-proteins that are often shared by multiple virus families thereby enabling preemptive drug development and therefore rapid deployment at the onset of an outbreak. Targeting host-pathways and cellular proteins that are hijacked by viruses can potentially offer broad-spectrum targets for the development of future antiviral drugs. Such host-directed antivirals are also likely to offer a higher barrier to the development and selection of drug resistant mutations. Given that most approved antivirals do not target host-proteins, we reinforce the need for the development of such antivirals that can be used in pre- and post-exposure populations.

## Background

The exponential global spread of SARS-CoV-2, the virus behind the COVID-19 pandemic, has stunned the world with a staggering socioeconomic and public health impact [1]. To date, this novel coronavirus has infected over 33 million people in 213 countries and resulted in over 1 million deaths worldwide [2]. Despite SARS-CoV-2 being the seventh known coronavirus to infect humans, the therapeutic landscape has remained barren, creating an urgent demand for the development of effective therapeutics for COVID-19 patients. While effective COVID-19 management requires both antiviral and anti-inflammatory treatment strategies, the need for a potent and safe antiviral for therapeutic and prophylactic use is undisputed. However, the expectations of developing safe and selective antiviral agents in a short time frame are impractical given that drug development from target discovery to approval takes 12 years on average [3]. Therefore, initial efforts have been focused on the repurposing of clinical stage or approved drugs. Even with therapeutic repurposing as a rapid strategy to redirect approved or clinical-stage drugs that were originally discovered for other diseases, the global community has witnessed the intricacies, nuances and challenges of drug development.

Of several approved drugs, in two separate in vitro studies, chloroquine (CQ) and hydroxychloroquine (HCQ) showed potent antiviral activity against SARS-CoV-2 [4]. Furthermore, a non-randomized, open label study in patients infected with the SARS-CoV-2 virus in Wuhan and other parts of China showed preliminary evidence of benefit against pneumonia and the clinical course of COVID-19 [5]. This initial excitement was tempered by two large, retrospective observational trials involving HCQ which were unable to demonstrate efficacy in COVID-19 patients [6, 7]. Moreover, the use of HCQ was associated with a significantly higher risk for in-hospital death, cardiac arrest, and QT interval prolongation, and other electrocardiogram  abnormalities [8–10]. As a result of these safety concerns, the use of HCQ for anti-COVID-19 management has diminished.

The approval of remdesivir, a nucleoside analog originally developed for the treatment Ebola virus infections, has added hope to the early management of COVID-19 infections. Remdesivir was found to have in vitro activity against SARS-CoV-2, further mechanism of action studies showed that it targets the viral nsp12 polymerase and acts as a chain terminator in viral replication. Recent clinical studies involving remdesivir have shown promising results as  its use is associated with a shorter time to recovery in comparison to placebo (11 days vs. 15 days) [11]. While remdesivir has received FDA approval for COVID-19 treatment, challenges with manufacturing and IV-administration have limited its widespread use. A key concept that is noteworthy from the above experience is the need for broad-spectrum antivirals with diverse mechanisms of action that are readily deployable for the prevention of future pandemics of known or unknown viruses.

## Direct-acting antiviral agents (DAAs) vs. host-directed antiviral agents (HDAs)

Most approved antiviral drugs target viral proteins, often acting selectively against one virus. Historically, drug development efforts have disproportionately focused on targeting viral proteins leading to the development of direct-acting antivirals (DAAs). However, viruses exploit numerous host proteins to carry out essential steps in their life cycles, and these proteins can be targeted for the development of host-directed antiviral agents (HDAs). Since viruses from one family often employ the same host proteins, targeting these proteins can produce agents with broad-spectrum antiviral activity and offer a higher barrier to the development of drug resistant virus strains. A key feature of HDAs is that their development can occur prior to the discovery of a new viral pathogen. The need for HDAs is underscored by the fact that there are over a dozen zoonotic viruses that have caused deadly human disease in recent years and will remain potential sources for future outbreaks. The last decade alone has witnessed two epidemics prior to COVID-19 in the form of the 2012 MERS epidemic (caused by another coronavirus) and the 2016 Zika epidemic (caused by an arthopod-borne flavivirus). A pre-existing repertoire of first-line, broad-acting HDAs that can be readily deployed may be beneficial in slowing the initial viral spread or in suppressing outbreaks. Later, HDAs can be complemented with DAAs and vaccines since their development hinges on the knowledge of specific viral proteins. While broad and deep investigation of viral-host pathways and targets is needed, the following examples illustrate a few of many cellular pathways that are utilized by different viruses, including coronaviruses, to replicate and cause infections.

### Host protease inhibition to restrict viral entry

Respiratory viruses such as influenza, parainfluenza and coronaviruses rely on host proteases for the activation of their entry factors that facilitate membrane fusion and entry into airway epithelial cells. The transmembrane protease serine 2 (TMPRSS2) is a ubiquitously expressed serine protease that is crucial to  the cleavage and activation of both, hemagglutinin (HA) of human influenza viruses and the spike (S) protein of SARS-like coronaviruses [12]. TMPRSS2 is dispensable for host development and homeostasis and thus may constitute an attractive therapeutic target [13]. Camostat, a clinical-stage serine protease inhibitor, is able to block viral entry of SARS-CoV-2 and influenza viruses [12]. Serine proteases involved with the pathogenesis of respiratory viruses are classified as trypsin-like proteases) which possess structurally conserved active site. This feature of trypsin-like proteases may be exploited for the design of inhibitors with broad-spectrum activity [14]. However, such targets are not without limitations as viruses often access molecular and biological redundancies in their host. Although camostat inhibited SARS-CoV-2 entry and replication, it did not completely abolish viral replication, likely reflecting residual S protein activation through alternative means. This is not surprising as the SARS-CoV-2 can also use the endosomal cysteine proteases cathepsin B/L to activate and prime its S protein in TMPRSS2 null cells. However, it must be noted that S protein processing by TMPRSS2, but not cathepsin B/L, is essential for viral entry [15, 16].

Moreover, the SARS-CoV-2 possesses a multibasic cleavage site which is processed by furin, another cellular protease. Furin induced pre-cleavage at the S1/S2 site likely promotes subsequent TMPRSS2-dependent entry into target cells. The presence of a furin-mediated cleavage site in viral proteins is often associated with highly pathogenic viral strains of influenza viruses [17]. Also, furin-mediated cleavage has been described for the processing of several viral glycoproteins across diverse viral families, including Borna-, Bunya-, Corona-, Filo-, Flavi-, Herpes-, Orthomyxo-, Paramyyxo-, Pneumo-, Retro- and Toga viruses [17]. In general, processing by furin can occur during viral production before egress from the producer cell or in the extracellular space during entry into target cells. Collectively, the presence of multiple exo- and endo- proteases offer drug design opportunities that may be efficiently accessed through drug combination strategies.

### Depletion of intracellular nucleotide pools and enhancement of viral mutagenesis

Viral replication places an increased cellular burden on the available nucleotide pools, which can be targeted to compromise viral replication. Inosine-5′-monophosphate dehydrogenase (IMPDH) catalyzes an essential step in the biosynthesis of guanine nucleotides, i.e., conversion of IMP to xanthosine monophosphate (XMP). XMP leads to the de novo formation of guanosine monophosphate (GMP), a crucial molecule for numerous cellular processes. Inhibition of IMPDH leads to depletion of intracellular guanine nucleotide (GTP /dGTP) levels and thus limits RNA and DNA synthesis needed for viral replication. Examples of IMPDH inhibitors are VX-497, a noncompetitive IMPDH-inhibitor with broad-spectrum activity [18], as well as ribavirin, which is a competitive IMPDH inhibitor.

In addition to dGTP-depletion, ribavirin also enhances viral mutagenesis by the incorrect substitution of ribaivirin triphosphate (RTP) in place of GTP into the viral RNA as most viral polymerases lack proofreading capacity [19]. However, despite a well-documented history of broad-spectrum antiviral activity, ribavirin displayed strikingly weak antiviral activity against SARS-CoV, MERS-CoV, and SARS-CoV-2 [20]. Coronaviruses possess a unique bifunctional enzyme called nsp14, which methylates the viral RNA cap and excises erroneous mutagenic nucleotides that are inserted by the error-prone nsp12 polymerase. This unique ability allows the coronavirus nsp14 to excise RTP from the viral genome limiting the antiviral activity of ribavirin. This extraordinary RNA correction machinery imparts nucleoside drug resistance to coronaviruses and is the likely source of their RNA-based genome expansion [21].

### Targeting pro-viral kinases and vesicular/secretory pathways

Recent genome-wide approaches using small interfering RNA (siRNA) or CRISPR assays targeting the cellular “kinome” have highlighted pro-viral cellular factors, which can serve as novel targets for the design of HDAs. As an example, de Wilde et al. performed a siRNA screen targeting the human kinome to identify host kinases relevant for SARS-CoV infection [22]. Their work also showed other proteins that promote SARS-CoV replication such as the coatomer protein complex (COPB2) and Golgi-specific brefeldin A resistant guanine nucleotide factor 1 (GBF1). Such examples underscore the importance of the vesicular and secretory pathways for viral replication. Similarly, Lesche et al. uncovered 133 genes required for the spread of multiple influenza virus strains. Further studies involving these target genes and 43 approved drugs showed that urea-based kinase inhibitors possess high antiviral activity and low cytotoxicity offering a superior therapeutic window. These inhibitors also showed substantial activity against other viruses such as cowpox virus (CPXV) and herpes simplex virus (HSV1) [23].

### Sigma receptors and virus-induced ER stress

Gordon et al. expressed 26 tagged, SARS-CoV-2 proteins in human cells to identify proteins that physically associated with each viral protein. They identified over 300 host proteins that bind to SARS-CoV-2 proteins, many of which are suspected of contributing to the viral life cycle. Among the interactors were Sigma1 and Sigma2 proteins. The authors tested 69 approved or clinical-stage compounds and found that the drug compounds targeting either the mRNA translation and/or regulators of Sigma1 and Sigma2 receptors displayed a prominent antiviral effect. Sigma1 receptors are localized at the endoplasmic reticulum (ER) membranes and the mitochondria-associated membranes (MAM). They are multifunctional proteins involved in essential cellular processes, including protein folding, degradation, ER trafficking and oxidative stress, cell survival, and mitochondrial function [24]. Sigma1 receptors have previously been implicated in the regulation of Hepatitis C [25] and Sendai virus replication via modulation of ER stress and the antiviral innate immune response [26]. Since multiple viruses induce ER stress, the Sigma1 receptor could be possible therapeutic target.

In addition to the above, modulation of epigenetic changes to the host genome [27], potentiation of immune responses and regulation of cytokine storms are also feasible strategies for the development of broad-spectrum, therapeutic agents. The latter two strategies have been extensively reviewed for COVID-19 management and are not discussed here.

## Current status and risk-benefit analysis of HDAs and DAAs

**Fig. 1:**
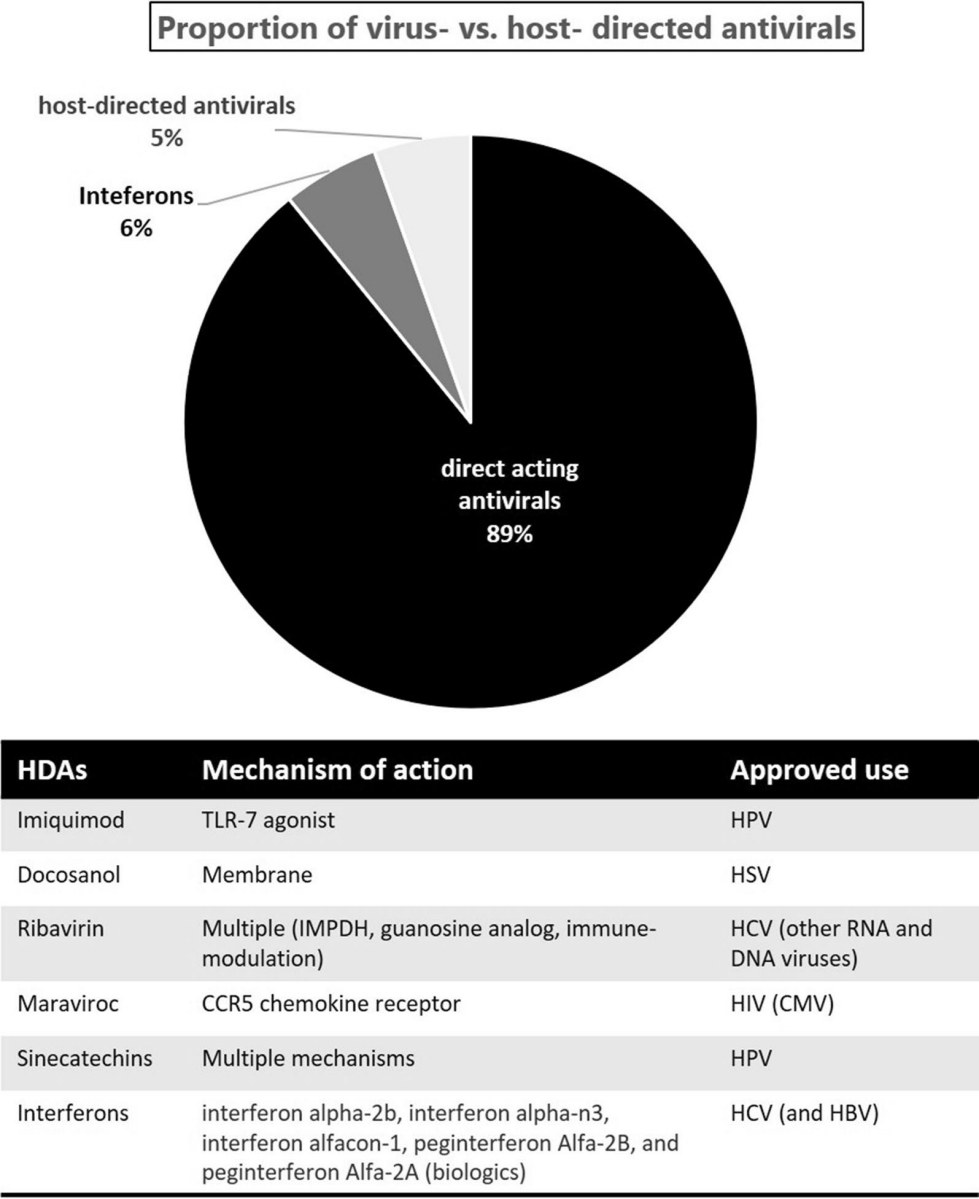
(Top) pie chart depicting the ratio of approved antiviral agents that are virus-directed vs. those that are host-directed. For clarification, interferons are represented as a separate class. (Bottom) list of approved HDAs and their mechanisms of action

Of the 92 approved antiviral drugs, HIV and HCV drugs account for two-thirds of all approvals [28]. The antiviral landscape is dominated by small molecules which constitute 87% of approved antiviral agents. Despite extensive studies involving host-targets and their relevance in the antiviral life cycle, the number of approved antivirals directed against host proteins has lagged significantly. Only about 10% of all approved antivirals are directed against host-proteins, half of which are interferon-related biologics [28, 29] (Fig. 1).

The clinical development of and application of an antiviral agent requires careful consideration of its putative benefit *vis a vis* its potential side-effects (Table 1). When targeting host proteins, the topic of drug-related adverse events (DAEs) and toxicity is inescapable. DAEs can be assigned to one of two sources i.e. chemical-related toxicities or pathway-related toxicities. The chemical toxicity of a potential drug is driven primarily by undesirable chemical liabilities of reactive, labile functional groups. On the other hand, pathway-related toxicity is a function of the biological pathway that is targeted for therapeutic intervention. While chemistry associated toxicities routinely encumber any drug development program, pathway-related toxicities are more complex. A common benefit of DAAs is that they are designed to hit a viral protein and not a host protein, thereby reducing theoretical concerns of off-target effects. However, even with DAAs, off-target effects are unavoidable as there are over tens of thousands of known host proteins.

**Table 1:** Potential advantages and disadvantages of HDAs (host targeted antivirals)

Benefits	Risks
Broad-spectrum activity against different virus types that use the same host target	Potential of host pathway-related toxicity
Pan-genotype/serotype coverage	Host/population-specific polymorphisms on differential host target expression
High barrier to the development of genetic resistance	Poor translation of in vitro to in vivo (animal models)
Numerous putative drug targets for cross-class combination therapy	Complex mechanism of action—Deconvolution of target and target-specific effects challenging
Can be available before epidemics and pandemics for emerging/new viruses	Possibility of redundant host mechanisms that ease virus dependence on select target
Potential for preemptive development of agents before a realized viral threat	May require direct-acting antivirals as a combination therapy for maximum benefit

For HDAs, such considerations must take center stage as targeting proteins or pathways that are important to cellular development and homeostasis should be avoided. The cardiac toxicity of HCQ in the COVID-19 setting effectively illustrates this point [8–10]. In the development of HDAs, the prospect of pathway-related toxicities will require more rigorous investigation in the preclinical and translational stages of drug development. These concerns must be tempered by the fact that such liabilities are routinely faced in the development of drugs for non-viral diseases where host proteins are consistently pursued, including diabetes, oncology and autoimmune diseases. Thus, modern drug development teams are adequately prepared to include such considerations during the mechanism of action and translational workup on a drug candidate.

Respiratory viral infections such as those caused by influenza and coronaviruses, or hemorrhagic fevers that are triggered by dengue or zika viruses with pandemic potential are acute infections that can resolve within a few weeks. Therefore, treatment strategies are characterized by short term use, permitting a higher threshold to accept non-fatal, adverse effects. It is noteworthy, that such risk-benefit analysis was recently employed by the FDA and the Data Safety Monitoring Committee in the ATCC-1 trial endorsing an emergency approval of remdesivir for COVID-19. While serious adverse effects (SAEs) were reported in 114 of the 541 (21%) patients in the remdesivir group, the approval suggests a higher tolerance for accepting SAEs in favor of the benefits associated with short term use.

## Conclusion

The SARS-CoV-2 pandemic underscores the need for both DAAs and HDAs in our antiviral armamentarium. Broad-spectrum antiviral agents such as HDAs can be readily deployed on a large scale to blunt viral spread while effective vaccines or DAAs are being developed. Although SARS-CoV-2 may fall short of triggering an apocalyptic scenario, it is an omen to looming viruses of known and unknown origins. Our antiviral drug development philosophy requires a careful reconsideration to include host-specific therapeutic targets for the management of viral infections.

## Data Availability

Not applicable.
